# Effects of dietary butyrate supplementation and oral nonsteroidal antiinflammatory drug administration on uterine inflammation and interval to first ovulation in postpartum dairy cows

**DOI:** 10.3168/jdsc.2022-0207

**Published:** 2022-06-17

**Authors:** L.E. Engelking, M. Gobikrushanth, M. Oba, D.J. Ambrose

**Affiliations:** 1 Department of Agricultural, Food and Nutritional Science, University of Alberta, Edmonton T6G 2P5, Canada; 2 Department of Large Animal Clinical Sciences, University of Saskatchewan, Saskatoon S7N 5B4, Canada

## Abstract

This study evaluated the effects of dietary butyrate supplementation and oral nonsteroidal antiinflammatory drug (NSAID) administration on uterine inflammation and the interval from calving to first ovulation (ICFO; in days). We hypothesized that a combination of dietary butyrate and oral NSAID would reduce uterine inflammation and decrease ICFO. Sixty-five cows were enrolled in a 2 × 2 factorial design and assigned to receive an iso-energetic diet containing a supplement of either butyrate (fatty acid-coated calcium butyrate) or control (commercial fat and calcium carbonate mixture) at 1.42% of diet dry matter, during the calving transition period from −28 (±3) to +24 (±3) days in milk (DIM; calving = d 0). At 12 to 24 h postcalving, cows received an oral NSAID (1 mg of meloxicam/kg of BW) or a placebo (food dye). Ovarian ultrasonography was performed weekly from 14 DIM until first ovulation or up to 56 DIM. Endometrial cytology was performed at 28 DIM to assess uterine inflammation based on polymorphonuclear leukocytes (PMN). No interactions were detected between treatments. The proportions of cows with high (>18%) endometrial PMN did not differ between butyrate and control diets or between NSAID and placebo. Mean (± standard error of mean) ICFO did not differ between butyrate (28 ± 2 d) and control (25 ± 2 d) or between NSAID (29 ± 2 d) and placebo (24 ± 2 d). However, the ovulation rate up to 56 DIM (hazard ratio: 0.61; 95% confidence interval: 0.35 to 1.04) established by survival analysis tended to be lower in NSAID than in placebo. In conclusion, dietary butyrate supplementation and oral NSAID administration did not reduce endometrial inflammation or the mean ICFO, but NSAID-treated cows tended to have a lower rate of ovulation up to 56 DIM.

## Highlights

• Dietary butyrate supplementation did not reduce endometritis or the mean interval to first ovulation.
• Giving oral NSAID treatment 12 to 24 hours after calving did not reduce endometritis or the mean interval to first ovulation but tended to lower the ovulation rate up to 56 days postpartum.
• Overall, neither dietary butyrate supplementation nor oral NSAID treatment reduced uterine inflammation or affected ovarian function in dairy cows.

## Graphical Abstract

**Figure:**
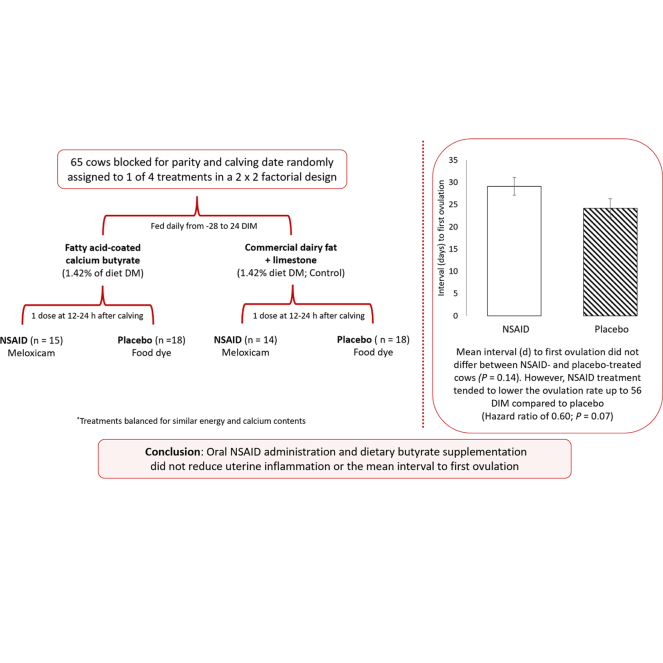
**Summary:** Cows experience postpartum uterine inflammation (endometritis), which may consequently delay ovulation. We evaluated whether the oral administration of a nonsteroidal antiinflammatory drug (NSAID; meloxicam) and the dietary supplementation of butyrate could reduce endometritis, determined by polymorphonuclear leukocytes (PMN), and reduce the interval to first ovulation. There were no differences between butyrate and control-fed cows for any of the parameters assessed. Mean interval to first ovulation did not differ between NSAID and placebo-treated cows, but NSAID-treated cows tended to have a lower ovulation rate up to 56 days postpartum compared with placebo-treated cows. Oral NSAID administration and dietary butyrate supplementation during the transition period were not effective in reducing endometritis and interval to first ovulation.

Postpartum dairy cattle regularly experience microbial invasion of the uterus, characterized by increased PMN in the uterine lumen (Kluciński et al., 1990). Although some uterine inflammation is necessary for homeorhetic adaption to lactation and return to estrus, dysregulated inflammation can result in endometritis (>18% PMN; Kasimanickam et al., 2004). Reproductive performance has been reported to decrease in cows with clinical (Pleticha et al., 2009) and subclinical endometritis (Kaufmann et al., 2009). Uterine inflammation has been suggested to delay return to ovarian cyclicity due to inhibited growth and function of the dominant ovarian follicle (Sheldon et al., 2002) and reduced reproductive hormones (Williams et al., 2007).

Short-chain fatty acids, such as butyrate, activate and regulate the immune system (Cox et al., 2009), and cows with lower concentrations of butyrate in utero-placental tissues are more likely to retain fetal membranes (Boro et al., 2014), which can predispose cows to endometritis (Potter et al., 2010). Therefore, adjusting dietary levels of short-chain fatty acids during the transition period may improve uterine health (Boro et al., 2014).

Parenteral administration of nonsteroidal antiinflammatory drugs (**NSAID**), such as acetylsalicylate, carprofen, and meloxicam, has been explored to manage endometritis, and NSAID have been reported to reduce inflammation (Pascottini et al., 2020) and increase pregnancy rate in dairy cattle (Priest et al., 2013). More recently, oral administration of meloxicam has been proposed to mitigate inflammation and pain in cattle (Shock et al., 2019) due to its relatively long half-life compared with injectable Metacam (Coetzee et al., 2009), and reportedly improved milk production (Carpenter et al., 2016). However, oral meloxicam has not been evaluated on uterine inflammation or ovarian function. Therefore, our objectives were to evaluate the effects of dietary butyrate supplementation and oral NSAID administration on uterine inflammation and the interval from calving to first ovulation in dairy cows (**ICFO**; in days).

This study was conducted at the University of Alberta Dairy Research and Technology Centre, a 146-cow tiestall barn (Edmonton, Alberta, Canada) in 2019–2020. All procedures were approved by the University of Alberta Animal Care and Use Committee for Livestock (AUP00003364). This experiment was conducted in tandem with Engelking et al. (2022).

Sixty-five (40 primiparous, 25 multiparous) Holstein cows were blocked by parity and expected calving date and randomly assigned to 1 of 2 iso-energetic diets containing fatty acid-coated calcium butyrate (1.0% butyrate, 0.24% calcium, and 0.18% fatty acids; Probiotech International) or a control supplement [1.04% commercial fat (Jefo Dairy Fat; 85% palmitic acid; Jefo Nutrition Inc.) and 0.38% calcium carbonate] at 1.42% of diet DM. Prepartum and postpartum diets, and details describing diet formulation and dietary butyrate concentration determination, are included in Engelking et al. (2022). Experimental diets were fed ad libitum through individual mangers at 0800 h, from 28 ± 3 d before expected calving date to 24 ± 3 d after calving. Day 1 was defined as the first day cows received experimental fresh cow diets.

Cows received an oral NSAID [meloxicam (15 mg/mL) suspension, USP; Solvet] administered at 1 mL/15 kg of BW in carrier solution, equivalent to 1 mg of meloxicam/kg of BW) or a placebo (food dye at 1 mL/15 kg of BW in carrier solution) at 12 to 24 h after calving using an oral drench gun. Study personnel were blinded to NSAID treatment until completion of data analysis. Treatment groups were (1) butyrate + NSAID (n = 15), (2) butyrate + placebo (n = 18), (3) control + NSAID (n = 14), and (4) control + placebo (n = 18).

Ovarian structures were evaluated once weekly by transrectal ultrasonography from 14 ± 3 d postpartum (DPP) until the first ovulation was confirmed, or until a maximum of 56 ± 3 d. Locations of major ovarian structures (follicles >5 mm diameter and luteal tissue) were recorded, and ovulation was confirmed by the appearance of a corpus luteum in the ovary. Vaginal mucus samples were collected using a Metricheck device (Simcro Tech Ltd.) at 14 and 28 d, and its appearance and odor were scored as per Williams et al. (2005) with the addition of an odor score category. Mucus appearance scores: clear or translucent = 0; white or off-white flecks of pus = 1; ≤50% white or off-white mucopurulent material = 2; and >50% purulent material, usually white or sanguineous = 3. Mucus odor scores: no odor = 0, faint nonfetid odor = 1, strong fetid odor = 2.

Samples for endometrial cytology were obtained at 28 d using a cytobrush (Medscand Medical) modified for use in large animals as described by Kasimanickam et al. (2004). In 3 of 65 cows (1 control + NSAID, 2 control + placebo), cytology samples could not be collected because the cervix was not passable. Cytological samples were smeared on microscope slides and fixed with cytofixative (Cytoprep, Fisher Scientific). Slides were then stained for a minimum of 8 min (Wright-Giemsa Stain; Fisher Scientific), washed with distilled water, dried, and examined under 400× magnification. Total cell and PMN counts were assessed to calculate % PMN based on at least 200 cells/slide. High and low PMN were defined as >18% and ≤18% PMN, respectively (Kasimanickam et al., 2004).

Statistical analyses were conducted using SAS (Statistical Analysis System, version 9.4 for Windows; SAS Institute Inc.). Normality of data was determined using the UNIVARIATE procedure. Binomial and continuous dependent variables were modeled against the fixed effects of independent variables (i.e., parity, dietary treatment, drug treatment, and their interactions) and analyzed using GLIMMIX procedure of SAS. For binomial dependent outcomes, the model was specified as binomial (dist = binary link logit), and the ilink option with Tukey's adjustment used to obtain corresponding least squares means by parity, dietary, and drug treatment groups. As none of the interactions was significant, the fixed effects of interactions were removed from the final model. The differences in the proportions of cows by vaginal mucus characteristics at 14 and 28 d were also analyzed by GLIMMIX procedure with Tukey's adjustment to obtain LSM by parity, dietary, and drug treatment groups. In addition to determining the proportion of cows that ovulated at 14, 21, 28, 35, 42, 49, and 56 DPP using the aforesaid GLIMMIX procedure, the probability of ovulation up to 56 d DPP was evaluated by the Kaplan-Meier survival analysis (LIFETEST procedure) and tested by a Cox proportional-hazards model (PHREG procedure). During data collection, 7 cows (butyrate + NSAID, n = 2; butyrate + placebo, n = 1; control + NSAID, n = 2; and control + placebo, n = 2) were treated with antibiotics or NSAID (other than the experimental treatment) for health disorders. Data for these cows were included up to when the above interventions occurred but removed from any statistical analysis after treatment. Significance was declared at *P* ≤ 0.05 and tendencies were declared when *P* > 0.05 but ≤0.10.

Contrary to our hypothesis, the proportions of cows with high (>18%) endometrial PMN did not differ by dietary (Figure 1; butyrate vs. control; 33 ± 9 vs. 35 ± 9%; *P* = 0.90) or drug treatment (NSAID vs. placebo; 43 ± 9 vs. 26 ± 9%; *P* = 0.17). The proportions of cows distributed by vaginal mucus characteristics on d 14 and 28 also did not differ (Table 1), but a smaller proportion of NSAID (3 vs. 17%) tended to have a vaginal mucus appearance score of 2 (≤50% mucopurulent material) at 28 DPP, and a larger proportion of NSAID had no vaginal mucus odor on d 28, compared with placebo (97 vs. 82%). While vaginal discharge can be associated with the growth of certain bacteria in the uterus (Williams et al., 2005), meloxicam reportedly does not affect uterine bacterial composition in early postpartum cows (Pascottini et al., 2021).

**Figure 1:**
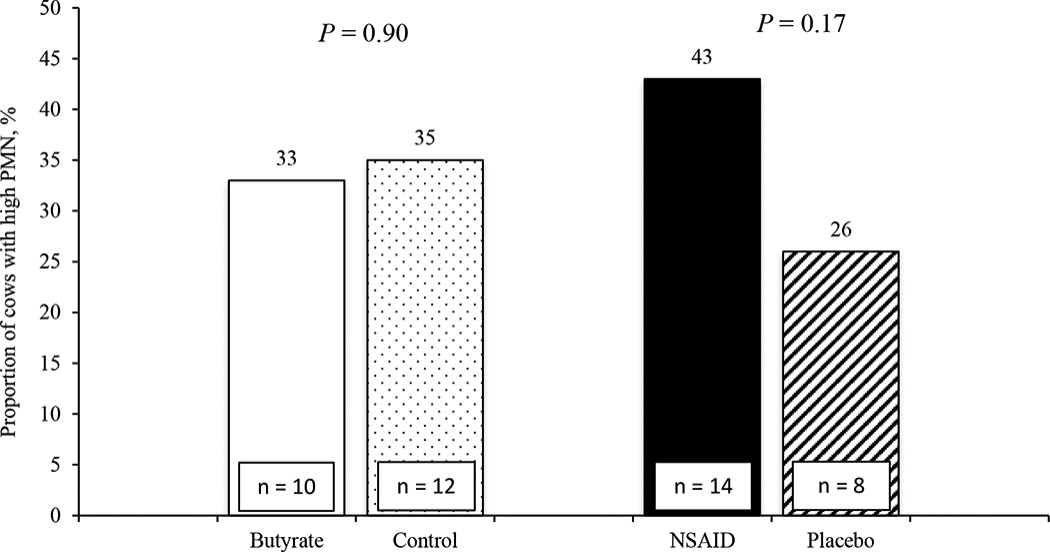
Proportions of cows with high uterine PMN (>18%; n = 22) by dietary butyrate [fatty acid-coated calcium butyrate supplement vs. control (commercial dairy fat and calcium carbonate supplement mixture) at 1.42% diet DM], and nonsteroidal antiinflammatory drug [1 mg of meloxicam/kg of BW vs. placebo (food dye)] treatment. High PMN was defined as >18% PMN based on endometrial cytology performed at 28 ± 3 DIM.

**Table 1:** Proportions (LSM %) of cows distributed by scores for vaginal mucus appearance (0, 1, 2, 3) and vaginal mucus odor (0, 1, 2) and by dietary^1^ (butyrate vs. control) or nonsteroidal antiinflammatory drug^2^ (NSAID vs. placebo) treatment at 14 and 28 d postpartum

Item	Vaginal mucus appearance score^3^				Vaginal mucus odor score^4^		
	0	1	2	3	0	1	2
d 14 postpartum							
Butyrate, % (n)	10 (3)	14 (4)	31 (9)	44 (13)	72 (20)	21 (7)	6 (2)
Control, % (n)	14 (5)	24 (8)	23 (8)	39 (15)	84 (29)	8 (4)	7 (3)
NSAID, % (n)	9 (3)	18 (6)	28 (9)	44 (15)	72 (23)	14 (6)	13 (4)
Placebo, % (n)	15 (5)	19 (6)	26 (8)	39 (13)	83 (26)	12 (5)	4 (1)
d 28 postpartum							
Butyrate, % (n)	31 (9)	59 (17)	5 (2)	3 (1)	95 (27)	3 (1)	1 (1)
Control, % (n)	29 (10)	44 (16)	10 (5)	11 (5)	89 (31)	9 (4)	1 (1)
NSAID, % (n)	31 (10)	58 (19)	3^a^ (1)	6 (3)	97^c^ (32)	2 (1)	0 (0)
Placebo, % (n)	29 (9)	44 (14)	17^b^ (6)	6 (3)	82^d^ (26)	10 (4)	7 (2)

a,b NSAID vs. placebo, *P* = 0.07.

c,d NSAID vs. placebo, *P* = 0.08.

1 Cows received butyrate (fatty acid-coated calcium butyrate supplement) or a control (commercial fat and calcium carbonate supplement mixture) at 1.42% of diet DM.

2 Cows received oral NSAID (1 mg of meloxicam/kg of BW) or placebo (food dye).

3 Mucus appearance scoring: 0 = clear or translucent; 1 = off-white or white flecks; 2 = ≤50% white or off-white mucopurulent material; and 3 = ≥50% purulent material, usually white or sanguineous (Williams et al., 2005).

4 Mucus odor scoring: 0 = no odor; 1 = faint odor; 2 = strong fetid odor (modified from Williams et al., 2005).

The cumulative proportions of cows that ovulated at each weekly interval (Figure 2) and the mean ICFO did not differ between butyrate and control (27.5 ± 2.1 vs. 25.3 ± 2.0 d; *P* = 0.44) or between NSAID and placebo (28.5 ± 2.0 vs. 24.3 ± 2.1 d; *P* = 0.14). The ovulation rate up to 56 DPP by survival analysis did not differ between butyrate and control (hazard ratio of 0.76; 95% CI 0.45 to 1.28; *P* = 0.30). However, the ovulation rate up to 56 DPP tended to be lower in NSAID than in placebo (hazard ratio of 0.61; 95% CI 0.35 to 1.04; *P* = 0.07). There were no differences in the interval from calving to the first detection of ovarian follicles of 10-mm diameter in butyrate compared with control (14.9 ± 0.7 vs. 15.1 ± 0.6 d; *P* = 0.76) or in NSAID compared with placebo (15.2 ± 0.6 vs. 14.8 ± 0.6 d; *P* = 0.63). Similarly, the interval from calving to the first detection of 16-mm diameter follicles did not differ in butyrate compared with control (19.4 ± 2.3 vs. 20.6 ± 2.2 d; *P* = 0.71) or in NSAID compared with placebo (20.8 ± 1.9 vs. 19.2 ± 2.6 d; *P* = 0.63). The absence of treatment differences in the intervals from calving to detection of 10- and 16-mm follicles indicates that the tendency for a lower ovulation rate up to 56 DIM in NSAID was not because of impeded recruitment of preovulatory size follicles.

**Figure 2:**
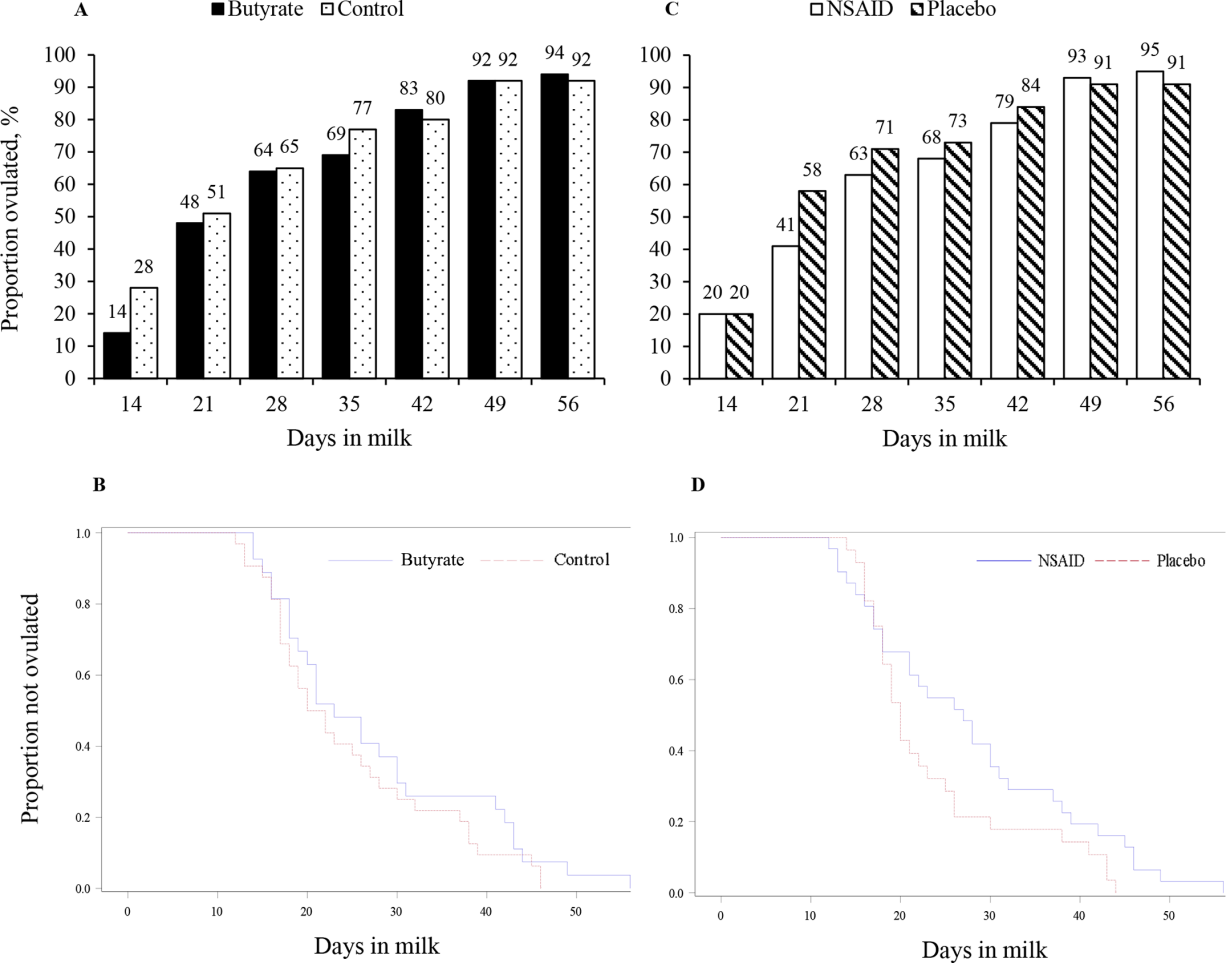
Proportions of cows ovulated by DIM given butyrate (fatty acid-coated calcium butyrate supplement; A, B) versus control (commercial fat and calcium carbonate supplement mixture) at 1.42% of diet DM, or when administered a nonsteroidal antiinflammatory drug (NSAID; 1 mg of meloxicam/kg of BW; C, D) versus placebo (food dye). Ovulation was detected by weekly transrectal ultrasonography and results were analyzed either by GLIMMIX procedure and plotted as bar charts (A, C), or by Kaplan-Meier survival analysis approach and plotted as survival function graphs (B, D). The proportions of cows that ovulated at each DIM did not differ between diet and drug treatments. The rate of ovulation up to 56 DIM did not differ between butyrate and control cows (hazard ratio: 0.75; 95% CI: 0.44 to 1.28; *P* = 0.29). The rate of ovulation up to 56 DIM tended to differ, with NSAID-treated cows having a lower hazard for ovulation compared with control cows (hazard ratio: 0.61; 95% CI: 0.35 to 1.04; *P* = 0.07).

Butyrate has been proposed to improve reproduction in cattle as it is an energy source (Ulfina et al., 2015); however, because rations were iso-energetic in the present study, additional energy from butyrate supplementation was unlikely. Overall, we did not observe any beneficial effects of supplemental butyrate.

Limited data are available on the effects of NSAID on uterine inflammation in dairy cattle; however, those who have assessed it found no changes (Priest et al., 2013; Meier et al., 2014; Pascottini et al., 2020), similar to our findings. Perhaps administration of NSAID 12 to 24 h postpartum was too early for the treatment to reduce inflammation at 28 DPP. Alternatively, it is possible NSAID administration, regardless of timing, does not affect endometrial PMN proportion. Additionally, Priest et al. (2013) found NSAID treatment for cows with subclinical endometritis improved pregnancy rate; thus, NSAID may be efficacious in cows with high PMN in the uterus (that is, those experiencing endometritis), but not necessarily in cows with low uterine PMN.

In the present study, NSAID was administered orally, whereas in much of the existing research NSAID was given through s.c. or i.m. routes. Although oral meloxicam has a similar therapeutic onset to the injectable form, the oral formulation has a longer half-life (27.5 h in calves) and a significantly longer lasting action (Coetzee et al., 2009). Injectable meloxicam may be cleared faster than oral meloxicam (14.33 vs. 3.20 h; Karademir et al., 2016); thus, the prolonged action of oral meloxicam may have interfered with natural postcalving inflammation, contributing to the tendency for delayed ovulation in NSAID. Some degree of uterine inflammation is required for uterine remodeling; thus, perhaps the initial treatment with NSAID delayed inflammation, resulting in postponed “rebound inflammation” (Farney et al., 2013), extending the duration of uterine recovery. This in turn may have reduced the ovulation rate in NSAID. This speculation is consistent with numerically greater proportion of high PMN cows in the NSAID group, but it must be noted that uterine inflammation was only assessed on d 28, and it was not significantly different between NSAID and placebo, so we cannot definitively say if this was a contributing factor.

We acknowledge that the differences in the modes of action of different NSAID formulations may have also contributed to the differences among studies. The present study used meloxicam, whereas other studies have evaluated carprofen (Priest et al., 2013; Meier et al., 2014) and sodium salicylate (Farney et al., 2013). Although it is possible that the lack of treatment effect on inflammation is due to timing, route of administration, or the type of NSAID, it is also possible that NSAID is not effective at mitigating endometrial inflammation in postpartum dairy cows.

Cows given the NSAID tended to have a reduced ovulation rate up to 56 DIM compared with cows given placebo in the present study. It has been reported that NSAID use delays ovulation in rodents (Gaytán et al., 2006) and humans (Sirois et al., 2004) primarily due to NSAID inhibition of prostaglandin. Increased endometrial PMN reportedly increases ICFO in cattle (Burke et al., 2010; Dourey et al., 2011; Green et al., 2011). This evidence is consistent with our findings that NSAID had numerically greater proportion (43 vs. 26%) of high PMN and slower rate of first ovulation up to 56 DIM. Cows with endometritis have impaired reproductive hormone production and slower ovarian follicle growth (Sheldon et al., 2010). Although reproductive hormones were not measured, ovarian follicular growth up to 10- and 16-mm diameter sizes was not affected in the present study, indicating that adequate gonadotropin support was available to sustain follicle growth and dominance.

It has been suggested that NSAID are likely more effective for cows experiencing calving difficulties (Laven et al., 2012) or inflammation, but when given to healthy cows, it may suppress inflammatory signaling in the immune system and lead to infections (Trimboli et al., 2020). Overall, it appears that there are risks associated with administering NSAID as a blanket treatment to all transition cows as inflammation is necessary to adapt to lactation; thus, it may be advisable to only provide NSAID to cows experiencing excessive inflammation following a difficult calving or other inflammatory conditions. As previously described, data used for analysis were obtained from cows without clinical diseases. The inclusion of only “healthy” cows may have reduced the efficacy of NSAID and could be a limitation of the present study. Another limitation is the lack of adequate statistical power in our study. Though originally planned with 120 cows, due to the limited availability of cows and for other reasons beyond our control, this was not possible.

In conclusion, neither dietary butyrate supplementation nor oral NSAID administration reduced endometrial inflammation or reduced the mean ICFO. However, NSAID-treated cows tended to have a lower ovulation rate up to 56 DPP than cows given placebo. Considering the lack of power in the present study, further research with a larger sample size is warranted to understand the effects of NSAID on uterine inflammation and ovarian function.
